# Decision Theory-Based COI-SNP Tagging Approach for 126 Scombriformes Species Tagging

**DOI:** 10.3389/fgene.2019.00259

**Published:** 2019-04-03

**Authors:** Cheng-Hong Yang, Kuo-Chuan Wu, Li-Yeh Chuang, Hsueh-Wei Chang

**Affiliations:** ^1^Department of Electronic Engineering, National Kaohsiung University of Science and Technology, Kaohsiung, Taiwan; ^2^Biomedical Engineering, Kaohsiung Medical University, Kaohsiung, Taiwan; ^3^Department of Computer Science and Information Engineering, National Kaohsiung University of Science and Technology, Kaohsiung, Taiwan; ^4^Department of Chemical Engineering and Institute of Biotechnology and Chemical Engineering, I-Shou University, Kaohsiung, Taiwan; ^5^Institute of Medical Science and Technology, National Sun Yat-sen University, Kaohsiung, Taiwan; ^6^Department of Medical Research, Kaohsiung Medical University Hospital, Kaohsiung Medical University, Kaohsiung, Taiwan; ^7^Department of Biomedical Science and Environmental Biology, Kaohsiung Medical University, Kaohsiung, Taiwan

**Keywords:** decision theory, DCST, single nucleotide polymorphism (SNP), barcoding, COI, teleost fish, species identification

## Abstract

The mitochondrial gene cytochrome c oxidase I (COI) is commonly used for DNA barcoding in animals. However, most of the COI barcode nucleotides are conserved and sequences longer than about 650 base pairs increase the computational burden for species identification. To solve this problem, we propose a decision theory-based COI SNP tagging (DCST) approach that focuses on the discrimination of species using single nucleotide polymorphisms (SNPs) as the variable nucleotides of the sequences of a group of species. Using the example of 126 teleost mackerel fish species (order: Scombriformes), we identified 281 SNPs by alignment and trimming of their COI sequences. After decision rule making, 49 SNPs in 126 fish species were determined using the scoring system of the DCST approach. These COI-SNP barcodes were finally transformed into one-dimensional barcode images. Our proposed DCST approach simplifies the computational complexity and identifies the most effective and fewest SNPs to resolve or discriminate species for species tagging.

## Introduction

The original concept of DNA barcoding was proposed to identify and discriminate a given species by a unique DNA sequence (Hebert et al., 2003). Such a DNA sequence aims at tagging species like a barcode. It is designed to identify a species from known DNA barcode sequences in a database. The commonly used DNA barcode of animal species is the mitochondrial gene cytochrome c oxidase I (COI) with a length of about 650 base pairs (bps). Meanwhile, COI sequences are also used for evolutionary and ecological studies (Hebert et al., 2003; DasGupta et al., 2005; Meier et al., 2006; Austerlitz et al., 2009; Kress et al., 2015; Park et al., 2018).

However, most nucleotides of the COI gene are conserved among different species except a minor proportion representing single nucleotide polymorphisms (SNPs). Several disease studies have used specific SNP to predict the predisposition for disease and the effects of therapeutic approaches. This concept has rarely been used for tagging species or improving the information content of DNA barcode sequences. The major benefit of using SNPs is the reduction of computational burden by removing the more abundant, non-informative, identical homologous nucleotides.

As an example, the tagging of fish species is not optimized as yet with respect to informative DNA barcoding. Some fish species have very similar morphology and it is difficult to distinguish those similar species, especially for marketing, conservation, and forensic purposes. Seafood mislabeling or fraud is a common societal and legal problem in fish trading (Sarmiento-Camacho and Valdez-Moreno, 2018) and the seafood economy (Vandamme et al., 2016; Willette et al., 2017). Currently, DNA barcoding is a reliable system for species identification and authentication and it is necessary to apply barcoding to many fish species (Liu et al., 2013; Vandamme et al., 2016; Willette et al., 2017; Sarmiento-Camacho and Valdez-Moreno, 2018). However, the COI sequences (~650 bp) are largely uninformative and too long for an optimized application for the above purposes.

In the present study, we follow the original concept of DNA barcoding to develop a decision theory-based COI SNP tagging (DCST) approach where only the variable nucleotides (SNPs) of a given COI barcode sequence is applied for the tagging of fish species. The Fish Barcode of Life Initiative (FISH-BOL) (Ward et al., 2009) provides a public database for DNA barcode sequences with images, and geospatial information for almost 10,000 fish species (Becker et al., 2011).

We use the idea of decision theory (Quinlan, 1986; Berger, 2013; Fernandez Slezak et al., 2018) to determine which sites (nucleotides) of DNA sequences are selected to discriminate between species. These are used to generate the unique DNA tags for classification. Using the DCST approach, SNPs are extracted from COI sequences to generate a SNP-based COI pattern. Finally, the SNP-COI pattern is transformed into a one-dimensional sequence barcode.

The major aim of our proposed DCST approach is to provide an effective identification tool by generating an SNP-COI barcode. Here we apply this to the example of 126 scombriform fishes.

## Materials and Methods

### Sampling and Data Pre-processing

We retrieved the COI sequences from 126 species of the bony fish (Teleostei) order Scombriformes that include representatives of the following families: Ariommatidae, Arripidae, Bramidae, Caristiidae, Centrolophidae, Chiasmodontidae, Gempylidae, Icosteidae, Nomeidae, Pomatomidae, Scombrinae, Scombrolabracidae, Scombropidae, Stromateidae, Tetragonuridae, and Trichiuridae. The sequence data, ranging from 648 to 685 base pairs (bp) in lengths, were obtained from GenBank. Details of the family name, species name, sequence length, and accession number are shown in Table 1. COI sequences (*n* = 126) from these scombriform fishes were aligned using the ClustalW tool in MEGA 7 software (Kumar et al., 2016). Subsequently, the 5′ and 3′ protruding sequences were trimmed to gain the same length of COI sequences.

**Table 1:** 126 COI sequences of the fish order Scombriformes from GenBank.

**Family**	**Genus**	**Species name**	**bp**	**Accession no**.	**Family**	**Genus**	**Species name**	**bp**	**Accession no**.
*Ariommatidae*	*Ariomma*	*bondi*	654	KT883659.1	*Scombrinae*	*Euthynnus*	*affinis*	652	DQ107685.1
*Ariommatidae*	*Ariomma*	*indica*	655	DQ107593.1	*Scombrinae*	*Grammatorcynus*	*bicarinatus*	655	DQ107676.1
*Arripidae*	*Arripis*	*georgianus*	655	EF609289.1	*Scombrinae*	*Grammatorcynus*	*bilineatus*	685	KF009597.1
*Arripidae*	*Arripis*	*trutta*	655	EF609290.1	*Scombrinae*	*Gymnosarda*	*unicolor*	652	JF493572.1
*Arripidae*	*Arripis*	*truttaceus*	655	KJ669393.1	*Scombrinae*	*Gasterochisma*	*melampus*	652	DQ107687.1
*Bramidae*	*Brama*	*brama*	655	EF609300.1	*Scombrinae*	*Katsuwonus*	*pelamis*	652	DQ107668.1
*Bramidae*	*Brama*	*dussumieri*	655	KF461140.1	*Scombrinae*	*Lepidopus*	*caudatus*	652	EU869824.1
*Bramidae*	*Brama*	*orcini*	652	KF489508.1	*Scombrinae*	*Rastrelliger*	*brachysoma*	652	DQ107680.1
*Bramidae*	*Pterycombus*	*brama*	652	KR086894.1	*Scombrinae*	*Rastrelliger*	*faughni*	655	KJ590069.1
*Bramidae*	*Pterycombus*	*petersii*	652	KF489737.1	*Scombrinae*	*Rastrelliger*	*kanagurta*	655	EF609587.1
*Bramidae*	*Taractes*	*asper*	652	GU440550.1	*Scombrinae*	*Scomber*	*australasicus*	652	DQ107708.1
*Bramidae*	*Taractichthys*	*longipinnis*	655	EF609476.1	*Scombrinae*	*Scomber*	*colias*	652	JQ774715.1
*Bramidae*	*Taractichthys*	*steindachneri*	655	EF609477.1	*Scombrinae*	*Scomber*	*scombrus*	652	DQ107718.1
*Bramidae*	*Xenobrama*	*microlepis*	655	EF609495.1	*Scombrinae*	*Scomberomorus*	*brasiliensis*	652	GU702363.1
*Caristiidae*	*Caristius*	*fasciatus*	652	KU176441.1	*Scombrinae*	*Scomberomorus*	*cavalla*	652	GU225658.1
*Caristiidae*	*Caristius*	*macropus*	652	GU440263.1	*Scombrinae*	*Scomberomorus*	*commerson*	652	DQ107670.1
*Centrolophidae*	*Centrolophus*	*niger*	655	EF609317.1	*Scombrinae*	*Scomberomorus*	*guttatus*	652	EF607533.1
*Centrolophidae*	*Hyperoglyphe*	*antarctica*	655	DQ107611.1	*Scombrinae*	*Scomberomorus*	*maculatus*	655	KF461233.1
*Centrolophidae*	*Hyperoglyphe*	*bythites*	655	KF461189.1	*Scombrinae*	*Scomberomorus*	*munroi*	652	DQ107660.1
*Centrolophidae*	*Hyperoglyphe*	*japonica*	652	JF952759.1	*Scombrinae*	*Scomberomorus*	*plurilineatus*	648	JF494457.1
*Centrolophidae*	*Hyperoglyphe*	*moselii*	652	DQ107609.1	*Scombrinae*	*Scomberomorus*	*queenslandicus*	652	DQ107653.1
*Centrolophidae*	*Hyperoglyphe*	*perciformis*	652	KC015488.1	*Scombrinae*	*Scomberomorus*	*semifasciatus*	655	DQ107654.1
*Centrolophidae*	*Hyperoglyphe*	*pringlei*	652	HQ945965.1	*Scombrinae*	*Scomberomorus*	*sierra*	652	GU440514.1
*Centrolophidae*	*Icichthys*	*lockingtoni*	652	GU440358.1	*Scombrinae*	*Sarda*	*australis*	652	DQ107712.1
*Centrolophidae*	*Lepidocybium*	*flavobrunneum*	652	EU752105.1	*Scombrinae*	*Sarda*	*orientalis*	655	EF609590.1
*Centrolophidae*	*Schedophilus*	*labyrinthicus*	655	EF609453.1	*Scombrinae*	*Sarda*	*sarda*	655	JQ623978.1
*Centrolophidae*	*Schedophilus*	*maculatus*	655	DQ107619.1	*Scombrinae*	*Thunnus*	*alalunga*	655	DQ107645.1
*Centrolophidae*	*Sarda*	*chiliensis*	652	EU752178.1	*Scombrinae*	*Thunnus*	*obesus*	655	DQ107629.1
*Centrolophidae*	*Seriolella*	*brama*	655	EF609461.1	*Scombrolabracidae*	*Scombrolabrax*	*heterolepis*	652	KJ768303.1
*Centrolophidae*	*Seriolella*	*caerulea*	655	EF609462.1	*Scombropidae*	*Scombrops*	*boops*	652	HQ945916.1
*Centrolophidae*	*Seriolella*	*punctata*	655	EF609463.1	*Stromateidae*	*Kali*	*indica*	651	EU148217.1
*Centrolophidae*	*Stromateus*	*brasiliensis*	652	EU074612.1	*Stromateidae*	*Pampus*	*argenteus*	655	DQ107596.1
*Chiasmodontidae*	*Chiasmodon*	*niger*	652	KY033590.1	*Stromateidae*	*Pampus*	*chinensis*	655	DQ107595.1
*Chiasmodontidae*	*Kali*	*normani*	652	GU440362.1	*Stromateidae*	*Pampus*	*cinereus*	652	EF607461.1
*Chiasmodontidae*	*Psenopsis*	*anomala*	652	EU595250.1	*Stromateidae*	*Pampus*	*echinogaster*	652	JN242665.1
*Chiasmodontidae*	*Psenopsis*	*cyanea*	655	EU392194.1	*Stromateidae*	*Pampus*	*punctatissimus*	652	JN242734.1
*Chiasmodontidae*	*Pseudoscopelus*	*astronesthidens*	652	KY033744.1	*Stromateidae*	*Peprilus*	*crenulatus*	652	KU201549.1
*Chiasmodontidae*	*Pseudoscopelus*	*lavenbergi*	652	MF957014.1	*Stromateidae*	*Peprilus*	*medius*	652	MF956931.1
*Gempylidae*	*Diplospinus*	*multistriatus*	652	KR086826.1	*Stromateidae*	*Peprilus*	*paru*	652	GU702367.1
*Gempylidae*	*Gempylus*	*serpens*	655	KF461182.1	*Stromateidae*	*Peprilus*	*simillimus*	652	GU440453.1
*Gempylidae*	*Nealotus*	*tripes*	652	KY033695.1	*Stromateidae*	*Peprilus*	*snyderi*	652	MF956937.1
*Gempylidae*	*Neoepinnula*	*orientalis*	652	GU804966.1	*Stromateidae*	*Peprilus*	*triacanthus*	652	KC015770.1
*Gempylidae*	*Nesiarchus*	*nasutus*	652	KR086867.1	*Stromateidae*	*Stromateus*	*fiatola*	648	JF494604.1
*Gempylidae*	*Promethichthys*	*prometheus*	662	KP244604.1	*Stromateidae*	*Stromateus*	*stellatus*	651	KY572905.1
*Gempylidae*	*Paradiplospinus*	*antarcticus*	652	KF930222.1	*Tetragonuridae*	*Tetragonurus*	*cuvieri*	655	DQ107601.1
*Gempylidae*	*Rexea*	*solandri*	649	LN907526.1	*Trichiuridae*	*Aphanopus*	*carbo*	652	KC015198.1
*Gempylidae*	*Scomber*	*japonicus*	652	EU752183.1	*Trichiuridae*	*Assurger*	*anzac*	652	GU440240.1
*Gempylidae*	*Thyrsites*	*atun*	652	JF494694.1	*Trichiuridae*	*Benthodesmus*	*simonyi*	652	JQ774573.1
*Icosteidae*	*Icosteus*	*aenigmaticus*	652	GU440359.1	*Trichiuridae*	*Benthodesmus*	*tenuis*	652	KF929659.1
*Nomeidae*	*Cubiceps*	*baxteri*	652	JF952712.1	*Trichiuridae*	*Evoxymetopon*	*poeyi*	651	JN990846.1
*Nomeidae*	*Cubiceps*	*gracilis*	652	KC015307.1	*Trichiuridae*	*Evoxymetopon*	*taeniatus*	651	JN990843.1
*Nomeidae*	*Cubiceps*	*pauciradiatus*	655	KJ968014.1	*Trichiuridae*	*Euthynnus*	*alletteratus*	652	GU225603.1
*Nomeidae*	*Cubiceps*	*whiteleggii*	655	DQ107602.1	*Trichiuridae*	*Kali*	*macrura*	651	EU148218.1
*Nomeidae*	*Nomeus*	*gronovii*	652	JF493993.1	*Trichiuridae*	*Lepidopus*	*altifrons*	652	KC015503.1
*Nomeidae*	*Psenes*	*arafurensis*	652	KT423112.1	*Trichiuridae*	*Lepturacanthus*	*roelandti*	651	JN990847.1
*Nomeidae*	*Psenes*	*maculatus*	652	KC015845.1	*Trichiuridae*	*Lepturacanthus*	*savala*	655	EF609540.1
*Nomeidae*	*Psenes*	*pellucidus*	655	DQ107607.1	*Trichiuridae*	*Scomberomorus*	*niphonius*	652	FJ238036.1
*Nomeidae*	*Psenes*	*sio*	652	MF957000.1	*Trichiuridae*	*Trichiurus*	*auriga*	669	KR105923.1
*Pomatomidae*	*Pomatomus*	*saltatrix*	655	DQ885110.1	*Trichiuridae*	*Trichiurus*	*brevis*	651	JN990852.1
*Scombrinae*	*Acanthocybium*	*solandri*	652	DQ107692.1	*Trichiuridae*	*Trichiurus*	*japonicus*	651	JN990868.1
*Scombrinae*	*Allothunnus*	*fallai*	652	DQ107703.1	*Trichiuridae*	*Trichiurus*	*lepturus*	652	EF607600.1
*Scombrinae*	*Brama*	*japonica*	652	FJ164426.1	*Trichiuridae*	*Trichiurus*	*nitens*	655	MF957079.1
*Scombrinae*	*Cybiosarda*	*elegans*	652	DQ107695.1	*Trichiuridae*	*Tentoriceps*	*cristatus*	651	JN990844.1

### Decision-Based COI SNP Tagging (DCST)

Decision theory (Berger, 2013) improves a decision-maker's choice among a set of alternatives that need to be considered. Most of decision theory is normative, prescriptive and descriptive that provides a decision that is completely rational, has perfect accuracy and easy understanding. Possible alternatives and outcomes are considered as follows: Step (1) clearly define the given problem, step (2) organize all the possible alternatives, step (3) be aware of all possible outcomes, step (4) consider the benefits of each alternative and outcome, step (5) create a mathematical decision theory rule model, and step (6) make a decision by evaluating the models.

Based on such understood decision making, we propose here an approach for DNA barcoding that generates shorter DNA barcodes. We here call a decision theory-based COI-SNP tagging (DCST) approach. Given an *N*×*M* matrix of sequence data, ***S*** is described as:

(1) $$\begin{array}{l} S\;=\;\left[\begin{array}{c} \begin{array}{c} \begin{array}{c} \begin{array}{c} s_{1,1}\\ s_{2,1} \end{array}\\ \begin{array}{c} \vdots \\ s_{N,1} \end{array} \end{array}\begin{array}{c} \begin{array}{c} s_{1,2}\\ s_{2,2} \end{array}\\ \begin{array}{c} \vdots \\ s_{N,2} \end{array} \end{array}\begin{array}{c} \begin{array}{c} s_{1,3}\\ s_{2,3} \end{array}\\ \begin{array}{c} \vdots \\ s_{N,3} \end{array} \end{array} \end{array}\begin{array}{c} \begin{array}{c} \begin{array}{c} \cdots \\ \cdots \end{array}\\ \begin{array}{c} \ddots \\ \cdots \end{array} \end{array}\begin{array}{c} \begin{array}{c} s_{1,M}\\ s_{2,M} \end{array}\\ \begin{array}{c} \vdots \\ s_{N,M} \end{array} \end{array} \end{array} \end{array}\right] \end{array}$$

where *N* is the number of sequences from each species and *M* is the nucleotide length. There are four nucleotide types A, T, G, and C in the matrix ***S***. Then the nucleotide frequency of distribution ***F*** is obtained in each position *p*ε [1, *M*]. The frequency distribution matrix ***F*** is represented by:

(2) $$\begin{array}{l} \text{F}\;=\left[\begin{array}{ccc} f_{A1}f_{A2}f_{A3} & \cdots & f_{AM}\\ f_{C1}f_{C2}f_{C3} & \cdots & f_{CM}\\ f_{G1}f_{G2}f_{G3} & \cdots & f_{GM}\\ f_{T1}f_{T2}f_{T3} & \cdots & f_{TM} \end{array}\right] \end{array}$$

where each frequency is calculated as follows:

(3) $$\begin{array}{l} f_{ip,\;i\in \left\{\text{A},\text{ C},\text{G},\text{ T}\right\}}=\overset{N}{\underset{k}{\sum }}\left(x_{k,\;p}|i\right) \end{array}$$

The decision rules are created to distinguish species and divide them with each step into two subgroups based on the score of each position of sequences. The calculation of score in each position is represented by:

(4) $$\begin{array}{l} \text{SCORE }=\;\left[\begin{array}{ccccc} score_{1} & score_{2} & {\text{score}}_{3} & \cdots & score_{M} \end{array}\right] \end{array}$$

where the estimated value at the position *p*, namely *score*_*p*_ is calculated as:

(5) $$\begin{array}{l} score_{p}\;=\;\frac{mid_{p}\;-\;diff_{p}}{mid_{p}}\;+\;weight_{p} \end{array}$$

where *mid*_*p*_ indicates the middle integer, i.e., the integer value of half of the number of sequence data (species number) in each subgroup,

(6) $$\begin{array}{l} mid_{p}\;=\;\left\lfloor \frac{\text{number of data set in node}}{2}\right\rfloor \end{array}$$

and *diff*_*p*_ is a parameter which balances the data for generating approximately equally sized subgroups. Therefore, biallelic loci with almost equal frequency for each allele get the highest scores and are selected to divide the data into 2 subgroups. The *mid*_*p*_ value is used to distribute all sequence data into two subgroups. For the equation for *diffp* (formula 7), our proposed methodology selects the first appearing SNP starting from the lowest to the highest order of nucleotide position although SNPs at different positions may have the same score. For example, there are four sequences in a given subgroup and the best case is that two data are assigned into the left subgroup and others are assigned to right subgroup. Accordingly, *diff*_*p*_ is calculated as (min denotes the minimum value):

(7) $$\begin{array}{l} diff_{p}\;=\underset{i\in \left\{A,\;C,G,\;T\right\}}{\min}\left\{\left|mid_{p}\;-\;f_{ip}\right|\right\} \end{array}$$

Moreover, two different nucleotide types make it easier to sort the sequences into two subgroups for tree construction. Three or four nucleotide types are complex and require more tree lineages. Accordingly, the logic of the weighting system (formula 8) of the DCST method emphasizes the two nucleotide types and assigns the highest score among them. Non-polymorphic loci are not considered in this method, and hence they are given a score of 0. The *weight*_*p*_ is defined by:

(8) $$\begin{array}{l} weight_{p}\;=\;\{\begin{array}{c} \begin{array}{c} 0,\text{ if the number of identified nucleotide type is 1}\\ 1,\text{ if the number of identified nucleotide types is 2} \end{array}\\ \begin{array}{c} 0.66,\text{ if the number of identified nucleotide types is 3}\\ 0.33,\text{ if the number of identified nucleotide types is 4} \end{array} \end{array} \end{array}$$

**Figure 1:**
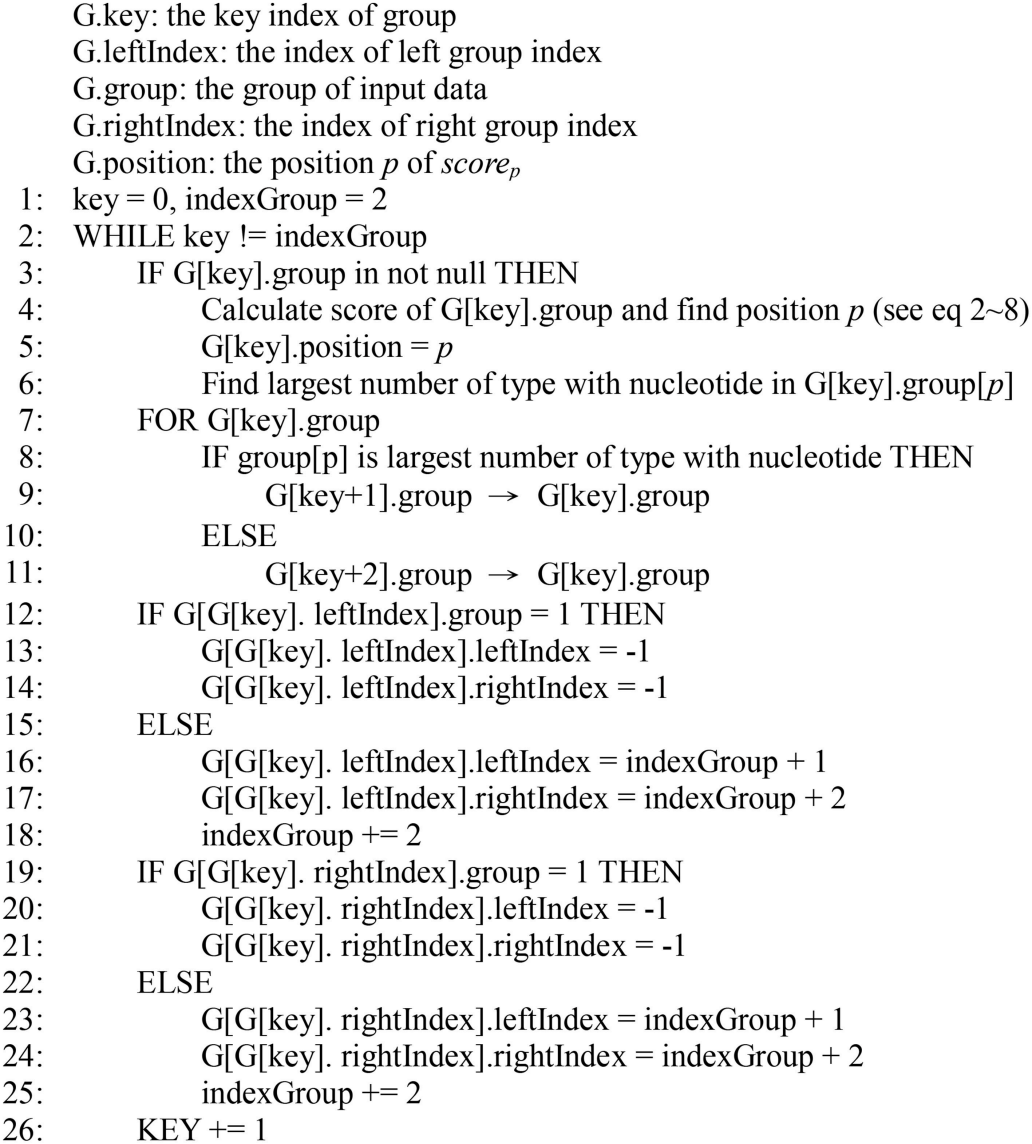
Pseudocode of the DCST approach.

The species can be separated into two subgroups according to the score estimation for each *score*_*p*_. The remaining subgroups at different levels are separated in the same way, and all the species are assigned a unique tag. The above step generates a pseudocode (Figure 1).

**Figure 2:**
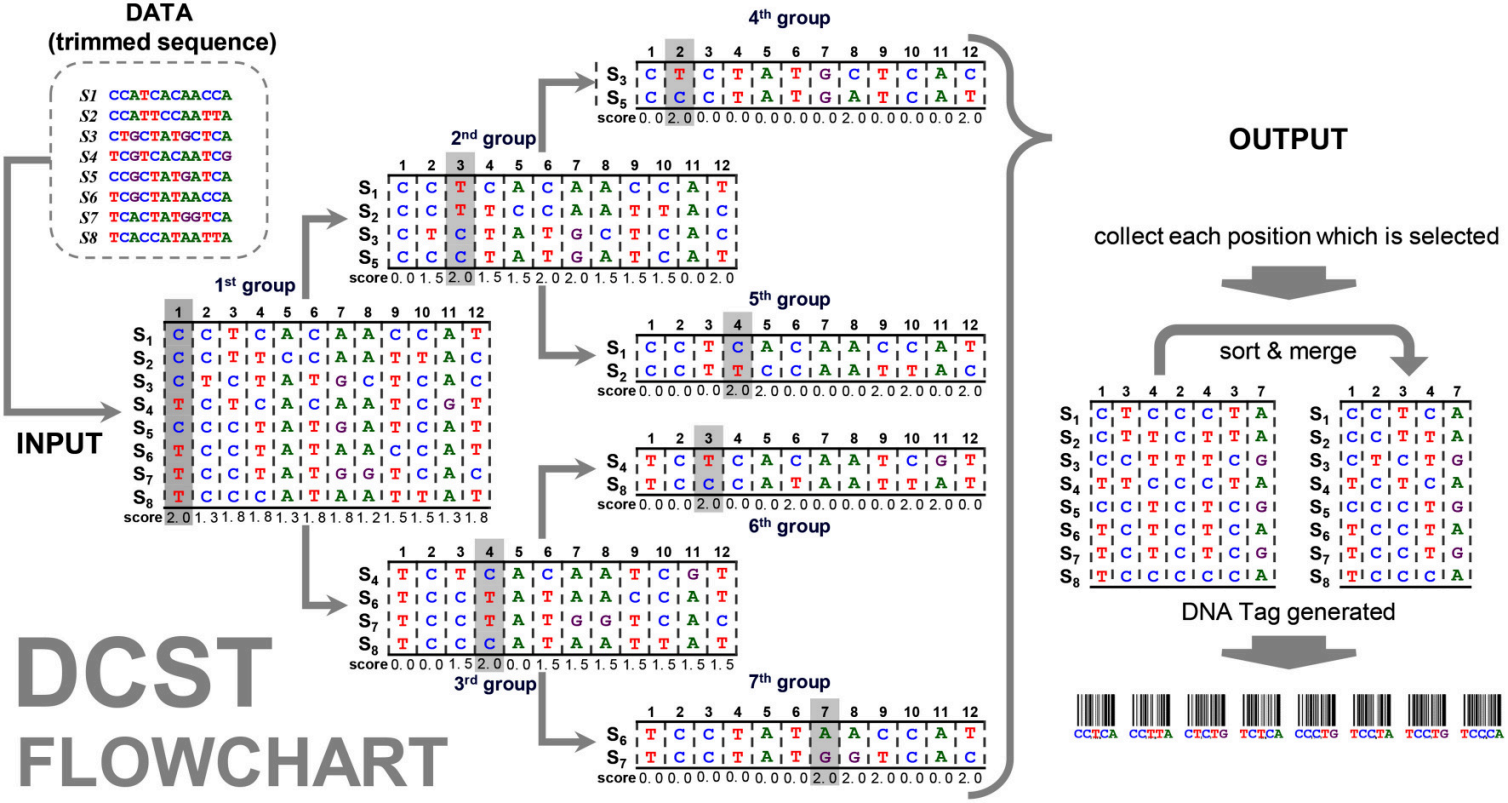
Flowchart of the DCST approach. This is an example to show how DCST approach operates. S1–S8 indicates eight sequences from eight species. In each level, the sequences are subgrouped according to the score system of DCSF approach, i.e., the nucleotides with the highest score are divided into two parts. Sometimes, the nucleotides at the same position may be chosen several times depending on the score performance.

The flowchart of the DCST approach is shown in Figure 2. For example, the “data” contain 8 sequences (species) with the length for 13 nucleotides. The frequency distribution ***F*** is counted from “data” (see formula 2 and 3) and the SCORE (*score*_*p*_) are calculated (see formula 4~8). The positions *p*_1_ and *p*_8_ at the first group has 8 sequences (species), therefore, the *mid*_1_ and *mid*_8_ are $\left\lfloor \frac{8}{2}\right\rfloor =4$ (formula 6) and the *diff*_1_ and *diff*_8_ are calculated as follows (formula 7):

$$\begin{array}{l} diff_{1}=\min\{\;\begin{array}{c} \begin{array}{c} f_{A1}=\left|4\;-\;0\right|\;=\;4\\ f_{C1}=\;\left|4\;-\;4\right|\;=\;0 \end{array}\\ \begin{array}{c} f_{G1}=\;\left|4\;-\;0\right|\;=\;4\\ f_{T1}=\;\left|4\;-\;4\right|\;=\;0 \end{array} \end{array}\;=\;0 \end{array}$$

and

$$\begin{array}{l} diff_{8}=\min\{\;\begin{array}{c} \begin{array}{c} f_{A8}=\;\left|4\;-\;6\right|\;=\;2\\ f_{C8}=\;\left|4\;-\;1\right|\;=\;3 \end{array}\\ \begin{array}{c} f_{G8}=\;\left|4\;-\;1\right|\;=\;3\\ f_{T8}=\;\left|4\;-\;0\right|\;=\;4 \end{array} \end{array}\;=\;2 \end{array}$$

where there are two types in *p*_1_ (C and T) and three types in *p*_8_, (A, C, and G) hence *weight*_1_ is 1 and *weight*_8_ is 0.66 (formula 8). The scores are calculated as follows (formula 5):

$$\begin{array}{l} score_{1}\;=\;\frac{4\;-\;0}{4}\;+\;1\;=\;2.0 \end{array}$$

and

$$\begin{array}{l} score_{8}\;=\;\frac{4\;-\;2}{4}\;+\;0.66\;≅\;1.2 \end{array}$$

This way we can get all scores of positions *p*_1_~*p*_8_, shown in Figure 2, and the maximum score in position *p*_1_ is calculated in the first group. All sequences are divided into subgroups with “up” and “down” sides as branches related to nucleotides (e.g., C and T). Then, the sub-group follows the same procedure as mentioned above until the end (i.e., 7th group). This way the positions *p*_1_, *p*_2_, *p*_3_, *p*_4_, and *p*_7_ are found. In this example, the positions, *p*_3_ and *p*_4_, are chosen twice, i.e., 2nd group/6th group and 3rd group/5th group. Therefore, much shorter informative barcode sequences become available using DCST.

Unique tags are generated when each species gets separated. Here, we use the code 128 (standard) of one dimensional barcodes to display each tag which is generated from a one dimension barcode image creator package called python-barcode 0.8.1. The standard code 128 in a one dimension barcode is an alphanumerical or numerical-only tool to generate barcode images.

## Results

### Retrieval of COI Sequences

**Figure 3:**
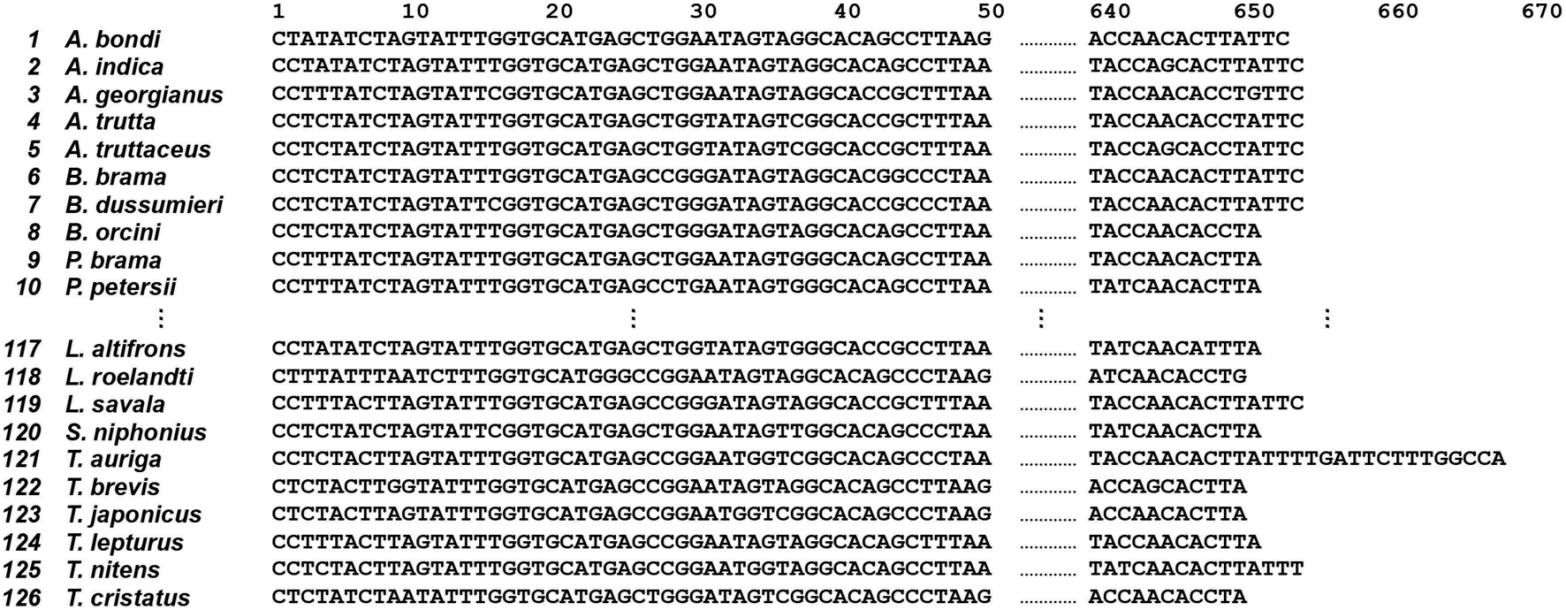
Original COI sequences (*n* = 126) of the fish order Scombriformes (Teleostei). This is an example of a group of species and sequences that shows 1st to 10th, 117th to 126th species and 1st to 50th, 640th to 668th position, respectively. The full original sequences for all species are available from http://shorturl.at/tBMVW.

In this study, we retrieved 126 COI sequences of the fish order Scombriformes from GenBank. The 126 original COI sequences are shown in Figure 3 (the full original data set is available at http://shorturl.at/ayEJ2).

### Alignment of COI Sequences

**Figure 4:**
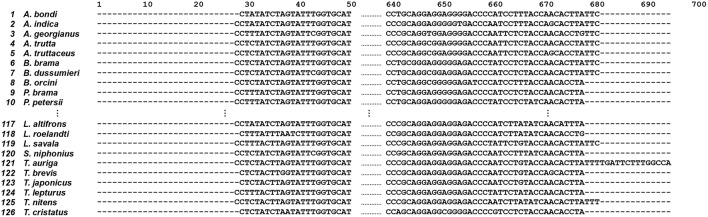
126 aligned COI sequences of the fish order Scombriformes (Teleostei). This is an example of a group of species and sequences that shows 1st to 10th, 117th to 126th species and 1st to 50th, 640th to 668th position, respectively. The full original sequences for all species are available from http://140.127.112.213/DNA_barcode/download/Scombriformes_COI_aligned.tar.

After performing multiple sequence alignments using the clustalW method in MEGA 7 software (Kumar et al., 2016), the resulting 126 aligned COI sequences are shown in Figure 4 (the full aligned data set is available at http://shorturl.at/tBMVW).

### Trimming of COI Sequences

**Figure 5:**
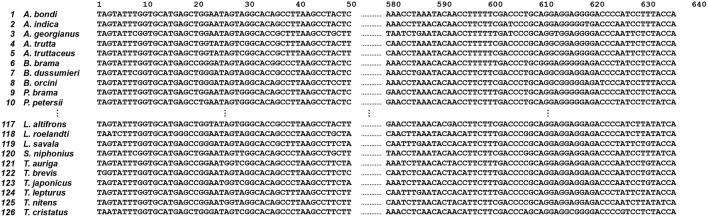
Trimmed COI sequences (*n* = 126) of the fish order Scombriformes (Teleostei). This is an ellipsis of part of species and sequences that shows 1st to 10th, 117th to 126th species and 1st to 50th, 580th to 636th position, respectively. The reference sequence listed at the top one of figure is derived from the accession number KT883659.1 for *A. bondi*. The 1st position of *A. bondi* at the top of this figure is the 8th position of KT883659.1 for *A. bondi*. The full original sequences for all species are available from http://140.127.112.213/DNA_barcode/download/Scombriformes_COI_trimmed.tar.

The position 1 to 35 and 673 to 696 of 126 aligned COI sequences are trimmed (i.e., protruding the 5′ and 3′ ends of sequence) that is shown as Figure 5 (the fully trimmed data set is available at http://shorturl.at/tTU04). Counting from the trimmed sequences, 281 SNPs were identified.

### Decision Process of COI Sequences

**Figure 6:**
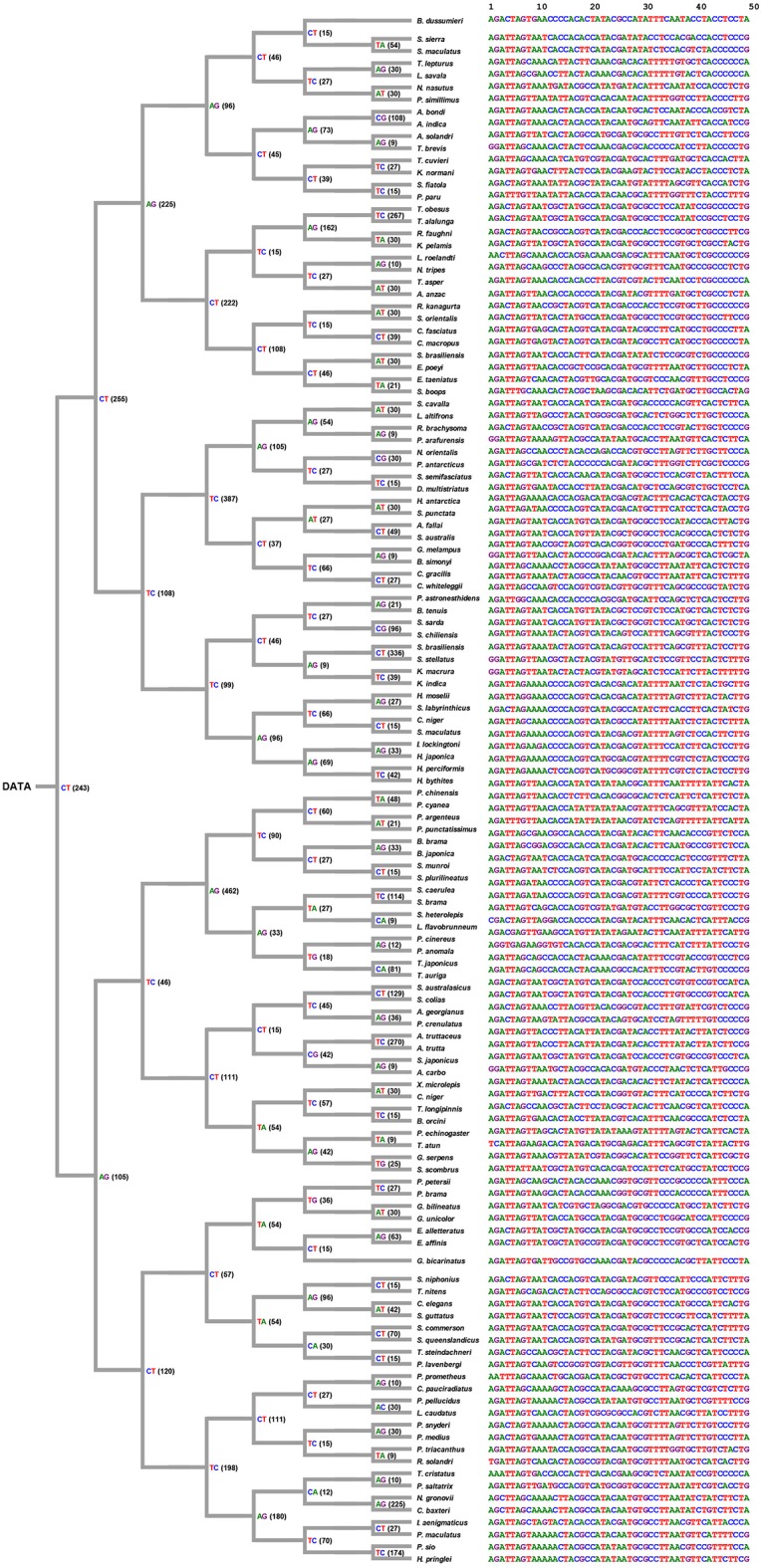
Tree-like structure outcome. This figure shows the selected position number and information of nucleotides for tagging SNP in 126 scombriform fishes. On the left side, the number of position within parentheses refers to the position of the reference sequence (*Ariomma bondi*; KT883659.1). For example, CT(243) indicates the nucleotide at the 243th position being selected as a node to separate two subgroups. It also shows the shorter tags from DNA COI sequences for each species on the right side. On the right side, the 1st nucleotide of the driftfish *A. bondi* has the 8th position in the original sequence KT883659.1 of *A. bondi*.

The decision process was created according the decision rule, and each unique tag was generated from each selected position (shown in Figure 6). Figure 6 shows *i*th position of nucleotides in each node, and all tags were collected and arranged from each node. Consequently, the original data of COI sequences with 636 bp length were curtailed into specific COI-SNP of only 49 bp length. Accordingly, our proposed DCST approach can effectively obtain shorter tags from COI sequences.

### Species-Tag Barcode Generation of COI Sequences

**Figure 7:**
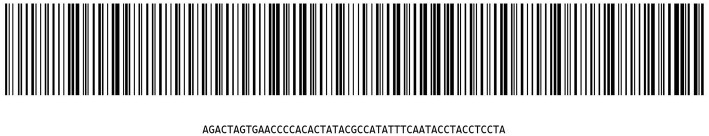
DNA tag barcode of *B. dussumieri*. As an example, a DNA tag barcode is generated for the purpose of fast and precise identification in the teleost goby *Boleophthalmus dussumieri*.

One-dimensional barcodes were generated from these unique tags (shown as Figure 7, the full tags of one dimensional barcodes for 126 scombriform species are available at http://shorturl.at/szJL1). These one-dimension barcode images of tags allow information retrieval with a barcode scanner for technical and scientific applications.

## Discussion

The original concept of “DNA barcoding” was thought to identify and discriminate between species by different genetic tags or markers. After a longer search for a most informative gene sequence, the mitochondrial COI gene was found to be most informative in animals at the species level. Besides for taxonomic identification purposes, it is commonly used recently in evolutionary and ecological studies (Hebert et al., 2003; DasGupta et al., 2005; Meier et al., 2006; Austerlitz et al., 2009; Kress et al., 2015).

Several applications of machine learning were developed in DNA barcoding taxonomy. For example, the BPSI2.0 interface program (Zhang and Savolainen, 2009) was developed by Zhang and collaborators which is based on back-propagation neural network for species identification. Weitschek et al. (2013) proposed a machine learning approach for species classification, called BLOG 2.0 (Barcoding with LOGic) which is based on character-based DNA barcode sequences. The supervised machine learning methods were later applied to DNA barcodes for species classification (Weitschek et al., 2014). They collected eight datasets of DNA barcode sequences and used four classifiers for classification analysis. The above approaches have in common, that the classification model builds up through a training data set, then it verifies testing data to assess the model performance.

However, our proposed DCST is different from the classification model “(Zhang and Savolainen, 2009; Weitschek et al., 2013, 2014) for which a for a large training data set of sequences is necessary to validate the model before it can be applied to the test data.” DCST arranges a short DNA barcode into a shorter DNA tag, which comes closer to the barcoding idea originally developed by Hebert et al. (2003). We propose here a DCST approach that generates an evolutionary COI-based identification system that provides even shorter sequences for the species tagging.

As for the decision rule of DCST, we will discuss two extreme cases caused by different designs. In case one, we search each position sequentially when a different nucleotide in *p*^th^ position is met the first time. This case shows a disordered outcome and indefinite rule leading to uncertainty or imbalance in the number of sequences in the branches of the trees (Figure S1). In case two, we search one of the nucleotides of maximum divergence in each position, its result shows a skewed outcome leading to imbalance tree (Figure S2). Although those two cases can generate unique DNA tags, they cannot segregate the sequence data for generating approximately equally sized subgroups. In contrast, the advantage of the balanced tree in algorithms and data structures area is the simple way to increase efficiency than other types of imbalance trees (Fleischer, 1996). In the present study, we used a balanced tree-based simple decision theory to arrange the species by COI barcoding systematically. Accordingly, the balanced tree algorithm DCST is theoretically more effective than the imbalanced tree methods (Figures S1, S2). Like the decision tree, the computational complexity time of DCST is *O*(N × M × D), where N is number of samples, M is the length of nucleotides, and D is the depth of tree (number of levels). Using 49 SNPs, the computational time for DCST to generate specific SNP species tags is 0.14693 ± 0.0016 s (mean ± SD; *n* = 30 runs) executed on an Intel Core i7-8750H 2.20GHz personal computer with 16 GB RAM. The length of sequences range from 648 bp to 685 bp which have approximately 4^650^ possible ATGC-combinations that would allow over 10 million species with unique DNA tags. Our proposed DCST method can, therefore, efficiently obtain shorter DNA barcode for species tagging. The obtained DNA tags can reduce data storage significantly compared to the full length COI sequence.

It is possible that multiple positions for *diff*_*p*_ (formula 7) may have the same score. For example, if there are 3 C, 3 T, and 2 A nucleotides in a node, the score is 1 or 2 where 3 C, 3 T, and 2 A = 8, i.e., *diff*_*p*_ = min for *mid*_*C*_–*f*_*Cp*_ = |$\lfloor \frac{8}{2}\rfloor -\;3$| = 1, *mid*_*T*_–*f*_*Tp*_ = |$\lfloor \frac{8}{2}\rfloor -\;3$| = 1, and *mid*_*A*_–*f*_*Ap*_ = |$\lfloor \frac{8}{2}\rfloor -\;3$| = 2. In this case, both C and T have the same score for selection and may be the candidates used for SNP barcoding. Both of them are theoretically suitable for the subsequent step of our proposed DCST method although different SNP barcode patterns may be generated. For convenience, the SNP is selected starting from the lowest to highest order of nucleotide position in the DCST method. Once the SNP is selected, then the procedure stops and goes to the next subgrouping process.

A limitation of the DCST approach for tagging species is that it is only used to discriminate the known species with known barcode sequences. However, DCST can still be applied to any other barcode sequence such as nuclear ribosomal internal transcribed spacer (ITS) (Seifert, 2009; Schoch et al., 2012) for fungi and ribulose-1,5-bisphosphate carboxylase/oxygenase (rubisco) and maturase K (matK) (Dong et al., 2014) for plants. Moreover, the DCST approach can be applied to the sequence data retrieved by Next Generation Sequencing (NGS). NGS offers high-throughput nucleotide sequencing for DNA/RNA molecules (Metzker, 2010). Recently, NGS has been applied to metagenomics (Roumpeka et al., 2017). NGS-profiling metagenomics may identify all species existing in a given environment. Using our proposed DCST approach, species-specific sequences may be processed to generate species-specific SNP barcodes for tagging species in metagenomics. Suitable SNPs from different positions are selected for species tagging in our proposed DCST system. However, the DCST system does not consider the distances between the selected SNPs. Therefore, the DCST system fails to calculate the evolutionary distance and is unsuitable for phylogenetic analysis. The tree generated in Figure 6 was just to demonstrate that the species in the collected data set have very close relationships with very similar sequences.

The practical application of this DCST system in a laboratory situation is to provide a platform for SNP arrays which allows fast and specific SNP genotyping. Here, SNPs belonging to COI-SNP based species-tags can be genotyped individually and simultaneously. These allow species identification by comparison with DCST-generated COI-SNP based species-tags. For example, Arrayed Primer Extension (APEX) is an array-based detection and can analyze thousands of SNPs in candidate region (Pullat and Metspalu, 2008). After processing to array scanner, the SNP pattern is generated and the species may be recognized immediately by checking the species-specific SNP pattern. In contrast, single gene PCR followed by sequencing needs a DNA sequencing machine and perform bioinformatics BLAST searching. Although both full sequence of a single locus and array assay of DCST-generated SNP can identify a species, DCST-generated SNP barcode is more suitable for species-tag barcode generation because few SNPs (~49 bp) are needed rather than full length of COI sequences (~650 bp). In other words, 49 SNPs only take 49 line codes but full length needs 650 line codes. Moreover, SNPs may spread out in different genes for the advanced species tagging in future. In this case, full length sequencing of different genes cannot be performed in the same reaction, however, array detection is allowed.

## Conclusion

The COI sequence with full length provides commonly accepted information for phylogenetic and evolutionary studies. However, the full length sequence contains mostly non-variable nucleotides and only a few SNPs. Our for the first time proposed DCST approach ignores the non-variable nucleotides by a scoring system and provides a format for the arrangement of SNP pattern for the identification of different fish species. This way we provide a decision-based COI SNP tagging (DCST) approach where the COI nucleotide sequence (~650 bp) is effectively reduced to a shorter COI-SNP barcode (49 bp) for the most informative discrimination of 126 scombriform fish species.

## Author Contributions

L-YC and H-WC conceived and designed the research and wrote the paper. C-HY instructed K-CW for algorithm processing. K-CW also contributed to sequence retrieval. C-HY and H-WC revised the paper. All authors read and approved the final manuscript.

### Conflict of Interest Statement

The authors declare that the research was conducted in the absence of any commercial or financial relationships that could be construed as a potential conflict of interest.

## Supplementary Material

Figure S1 Sequential searching for SNP is designed to subgroup the COI sequences at each level. In this case (case I), sequential searching is designed to find the diallelic type of SNP at each homologous position and perform subgrouping based on alternative nucleotides at this SNP. However, this case does not consider the nucleotide distribution compared to our proposed DCST method. For example, we found the nucleotide at the first position (nt 1) was a SNP and these sequences were separated into two subgroups based on this SNP (T/C) at 1-level, i.e., S_1_, S_2_, S_4_ (T) are allocated to the top side and S_3_, S_5_, S_6_, S_7_ (C) are allocated to the bottom side. In the top side of 2-level, the second nucleotide (nt 2) is not a SNP and is skipped. Then, the third nucleotide (nt 3) is a SNP and these sequences were separated into two subgroups based on this SNP (C/T) at 2-level, i.e., S_2_ (C) are allocated to the top side and S_1_ and S_4_ (C) are allocated to the bottom side. Subgrouping for the other levels follows the same rule as mentioned above.

Figure S2 Unique searching for SNP is designed to subgroup the COI sequences at each level. In this case (case II), unique searching is designed to find the SNP with only unique nucleotide for one unique subgroup and the other sequences are processed for next unique searching. For example, the first nucleotide (nt 1) does not show one unique nucleotide, i.e., 3 T and 5 C. Subsequently, the unique searching goes to the second nucleotide. We found the second position (nt 2) of S3 (T) is unique compared to others (C) at the 1-level, i.e., S3 (T) is allocated to the top side and others (C) are allocated to the bottom side. Subgrouping for the other levels follows the same rule as mentioned above.
