# Supplementary figures and images for: Decision Theory-Based COI-SNP Tagging Approach for 126 Scombriformes Species Tagging
Source: Front Genet. 2019 Apr 3;10:259. doi: 10.3389/fgene.2019.00259 (PMC6456664; doi:10.3389/fgene.2019.00259)

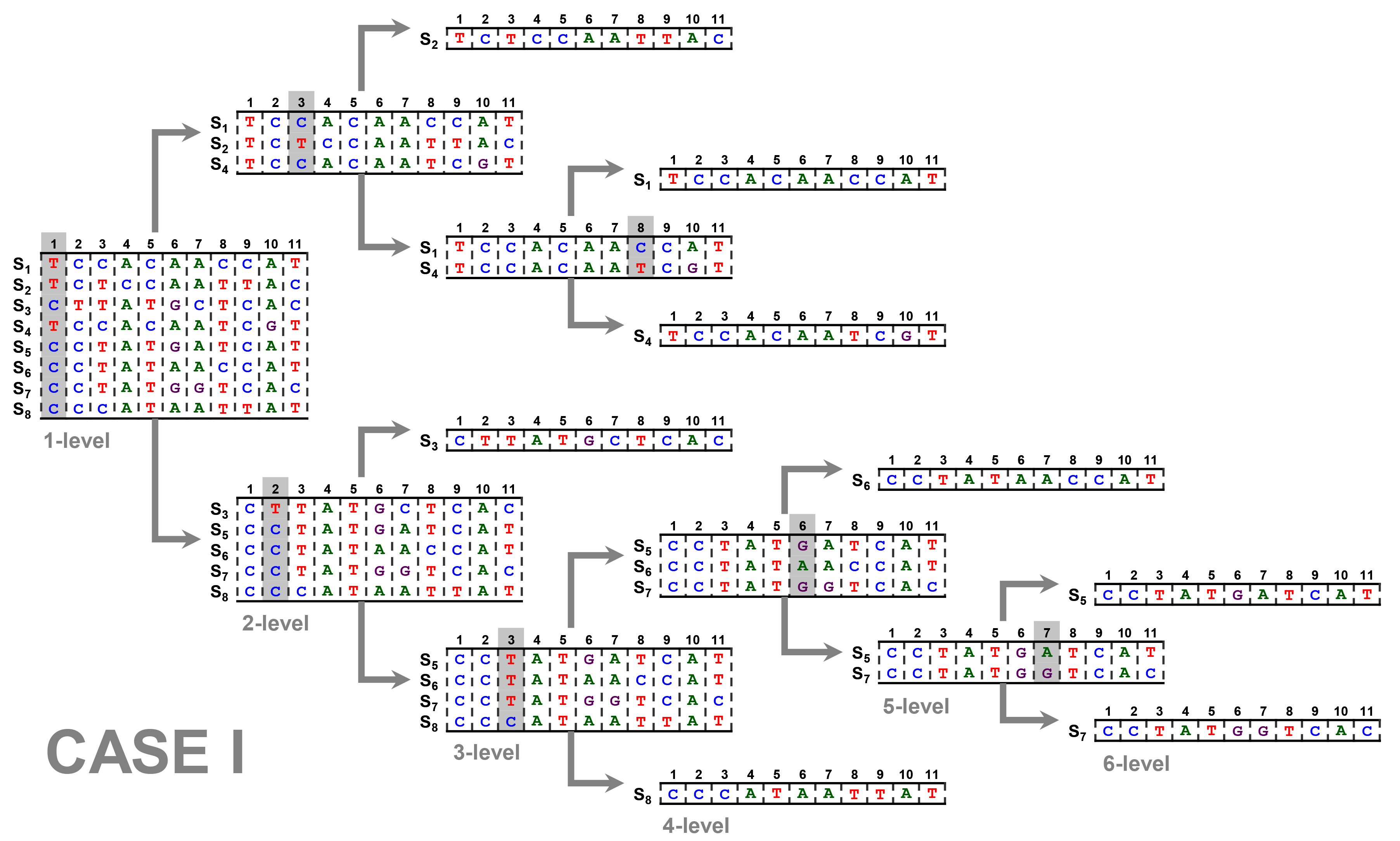

Supplement: Figure S1 — Sequential searching for SNP is designed to subgroup the COI sequences at each level. In this case (case I), sequential searching is designed to find the diallelic type of SNP at each homologous position and perform subgrouping based on alternative nucleotides at this SNP. However, this case does not consider the nucleotide distribution compared to our proposed DCST method. For example, we found the nucleotide at the first position (nt 1) was a SNP and these sequences were separated into two subgroups based on this SNP (T/C) at 1-level, i.e., S1, S2, S4 (T) are allocated to the top side and S3, S5, S6, S7 (C) are allocated to the bottom side. In the top side of 2-level, the second nucleotide (nt 2) is not a SNP and is skipped. Then, the third nucleotide (nt 3) is a SNP and these sequences were separated into two subgroups based on this SNP (C/T) at 2-level, i.e., S2 (C) are allocated to the top side and S1 and S4 (C) are allocated to the bottom side. Subgrouping for the other levels follows the same rule as mentioned above. [file Image_1.TIF]

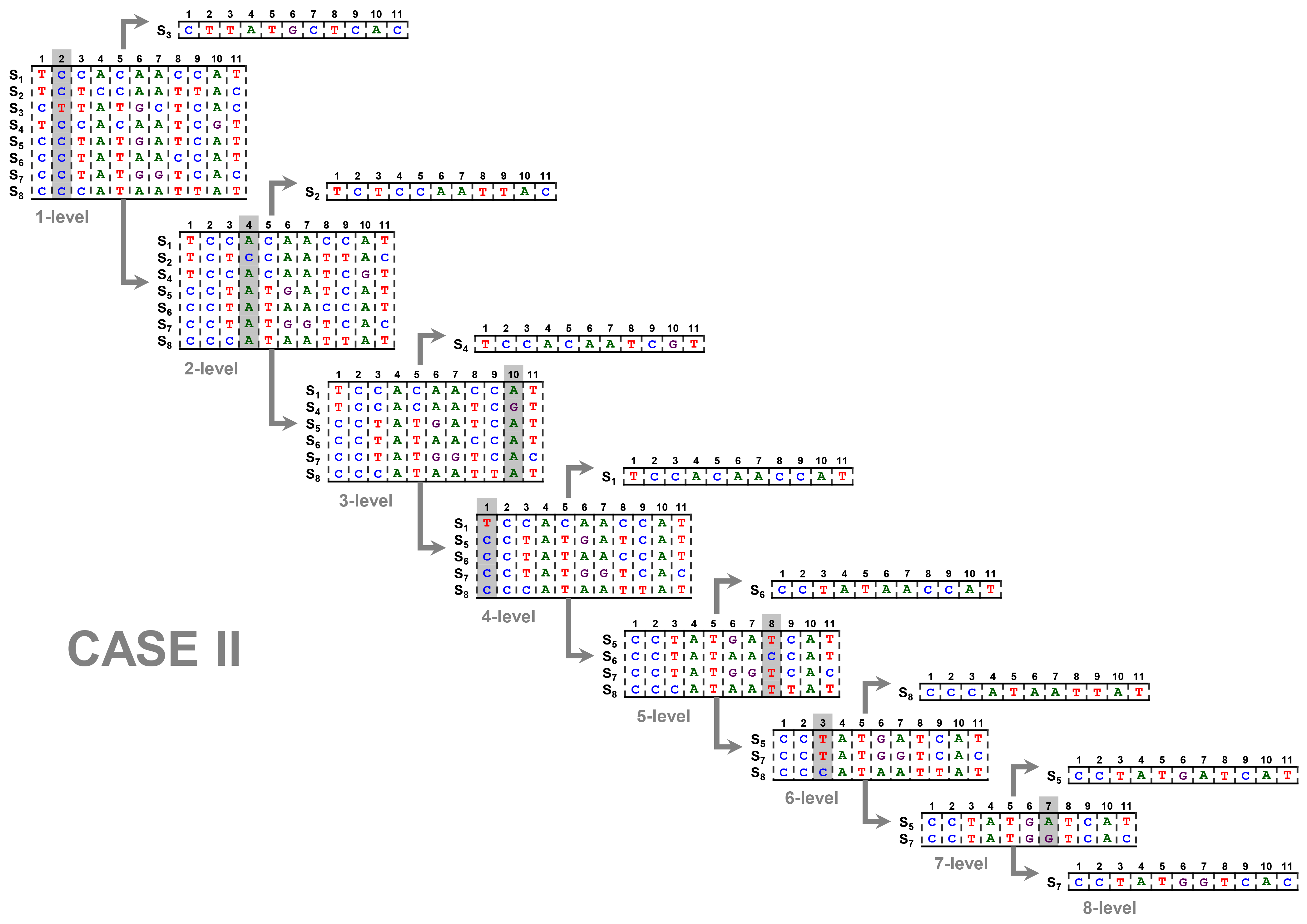

Supplement: Figure S2 — Unique searching for SNP is designed to subgroup the COI sequences at each level. In this case (case II), unique searching is designed to find the SNP with only unique nucleotide for one unique subgroup and the other sequences are processed for next unique searching. For example, the first nucleotide (nt 1) does not show one unique nucleotide, i.e., 3 T and 5 C. Subsequently, the unique searching goes to the second nucleotide. We found the second position (nt 2) of S3 (T) is unique compared to others (C) at the 1-level, i.e., S3 (T) is allocated to the top side and others (C) are allocated to the bottom side. Subgrouping for the other levels follows the same rule as mentioned above. [file Image_2.TIF]
